# Correlation of organelle interactions in the development of non-alcoholic fatty liver disease

**DOI:** 10.3389/fimmu.2025.1567743

**Published:** 2025-04-16

**Authors:** Jiabao Liao, Mengqiu Shao, Ze Zhou, Si Wang, You Lv, Yanming Lu, Fang Yao, Wenting Li, Ling Yang

**Affiliations:** ^1^ First Clinical Medical College, Yunnan University of Chinese Medicine, Kunming, Yunnan, China; ^2^ Department of Endocrinology, Jiaxing Hospital of Traditional Chinese Medicine, Jiaxing, China

**Keywords:** organelles, interactions, MCSs, NAFLD, ER

## Abstract

Organelles, despite having distinct functions, interact with each other. Interactions between organelles typically occur at membrane contact sites (MCSs) to maintain cellular homeostasis, allowing the exchange of metabolites and other pieces of information required for normal cellular physiology. Imbalances in organelle interactions may lead to various pathological processes. Increasing evidence suggests that abnormalorganelle interactions contribute to the pathogenesis of non-alcoholic fatty liver disease (NAFLD). However, the key role of organelle interactions in NAFLD has not been fully evaluated and researched. In this review, we summarize the role of organelle interactions in NAFLD and emphasize their correlation with cellular calcium homeostasis, lipid transport, and mitochondrial dynamics.

## 1 Introduction

Organelles such as the endoplasmic reticulum (ER), mitochondria, and lipid droplets engage in highly coordinated information exchange and material transfer, forming an intracellular organelle interaction network (1). Normal intracellular interactions are beneficial for maintaining the stability between organelles and various physiological and biochemical reactions. Abnormal intracellular interactions can lead to a series of diseases such as cardiovascular diseases, cancer, diabetes, and metabolic disorders (2).

Changes in people’s dietary structure and lifestyle coming with the development of the social economy have led to an increasing incidence of non-alcoholic fatty liver disease (NAFLD), which has become the most common chronic liver disease globally (3). The widespread prevalence of NAFLD directly leads to an increase in the incidence of cirrhosis, liver cancer, and cardiovascular diseases, posing a significant threat to human health and brings a heavy economic burden to patients, families, and society (4). Currently, the pathogenesis of NAFLD is not fully understood; however, in recent years, organelle interactions have attracted widespread attention in the occurrence and development of NAFLD. These interactions are closely related to lipid metabolism abnormalities, cellular calcium imbalance, and abnormalities in mitochondrial dynamics, all of which are potential mechanisms of NAFLD. In this review, we discuss the key role of organelle interactions in NAFLD.

## 2 Interactions between organelles

### 2.1 Interactions exist between organelles

In eukaryotic cells, there are various subcellular structures enclosed by single or double membranes—organelles, such as the ER, mitochondria, lipid droplets, Golgi apparatus, and lysosomes. Organelles may appear relatively independent, each with specific biological functions. In reality, these organelles often come into contact with each other, maintaining normal cell function through fine division of labor and collaboration (5). As one of the largest organelles in eukaryotic cells, the ER is responsible for the synthesis, folding, modification, structural maturation, and targeted distribution of proteins (6, 7), and it is also the center of lipid metabolism (6). Mitochondria are the main regulators of energy metabolism (8). They have two layers of membranes (9). In addition, they possess a circular genome known as mitochondrial DNA (mtDNA) (9). The Golgi apparatus, located near the cell nucleus, is a membrane-bound organelle. It primarily handles the transport, processing, and sorting of membrane proteins, secretory proteins, and lipids (10). Peroxisomes play various important roles in different cells. These functions include fatty acid β-oxidation, hydrogen peroxide degradation, glycerol metabolism, and the maintenance of cellular integrity (11). Lipid droplets (LDs),which are mainly located in the cytoplasm, store neutral lipids for energy or membrane synthesis (12). Early endosomes are carriers that enable the plasma membrane to absorb extracellular substances and can transport these substances to the plasma membrane, ER, or late endosomes (12). The digestive action of lysosomes can obtain extracellular or cell surface cargo through endocytosis, and also degrade intracellular components through autophagy (12). Endosomes and lysosomes work together in endocytosis and degradation, forming the endosomal system (13, 14).

### 2.2 Organelle interactions are facilitated through membrane contact sites

Organelle interactions are achieved through tiny membrane connections formed between them, which are known as membrane contact sites (MCSs) (15). Although the membranes from two organelles are in close contact at MCSs, they maintain their distinct identities and do not fuse. MCS formation shortens the distance between organelles, and this distance can vary within a certain range. For example, in mammalian cells, the distance between the ER and the plasma membrane dynamically varies between 19 nm and 22 nm (16). In budding yeast, this range is 17–57 nm (17). Additionally, MCSs can either dynamically change or remain stable depending in accordance with the requirements of cellular functionality.

### 2.3 Organelles complete important physiological functions through interactions

Observations through electron microscopy and fluorescence microscopy indicate that the structure of MCSs is relatively stable, indicating that connections between organelles have important functions (18). For example, the inositol trisphosphate receptor on the ER membrane binds with channel proteins and glucose-regulated protein 75 on the mitochondria to form MCSs, regulating calcium ion transport and maintaining mitochondrial calcium homeostasis (15). The actin nucleation factor at the contact points between the ER and mitochondria promotes the formation of actin complexes, recruits dynamin-related protein 1 to the outer mitochondrial membrane, and facilitates mitochondrial contraction and fission (19, 20). Research indicates that mitochondrial fission occurs at the junctions of mitochondria, endoplasmic reticulumER, and lysosomes, where Ras-related GTP-binding protein 7 promotes the formation of MCSs between lysosomes and mitochondria, participating in mitochondrial division (21). Other studies (22) have demonstrated that interactions between the ER and mitochondria are also closely related to mitochondrial fusion.

## 3 ER network

**Figure 1:**
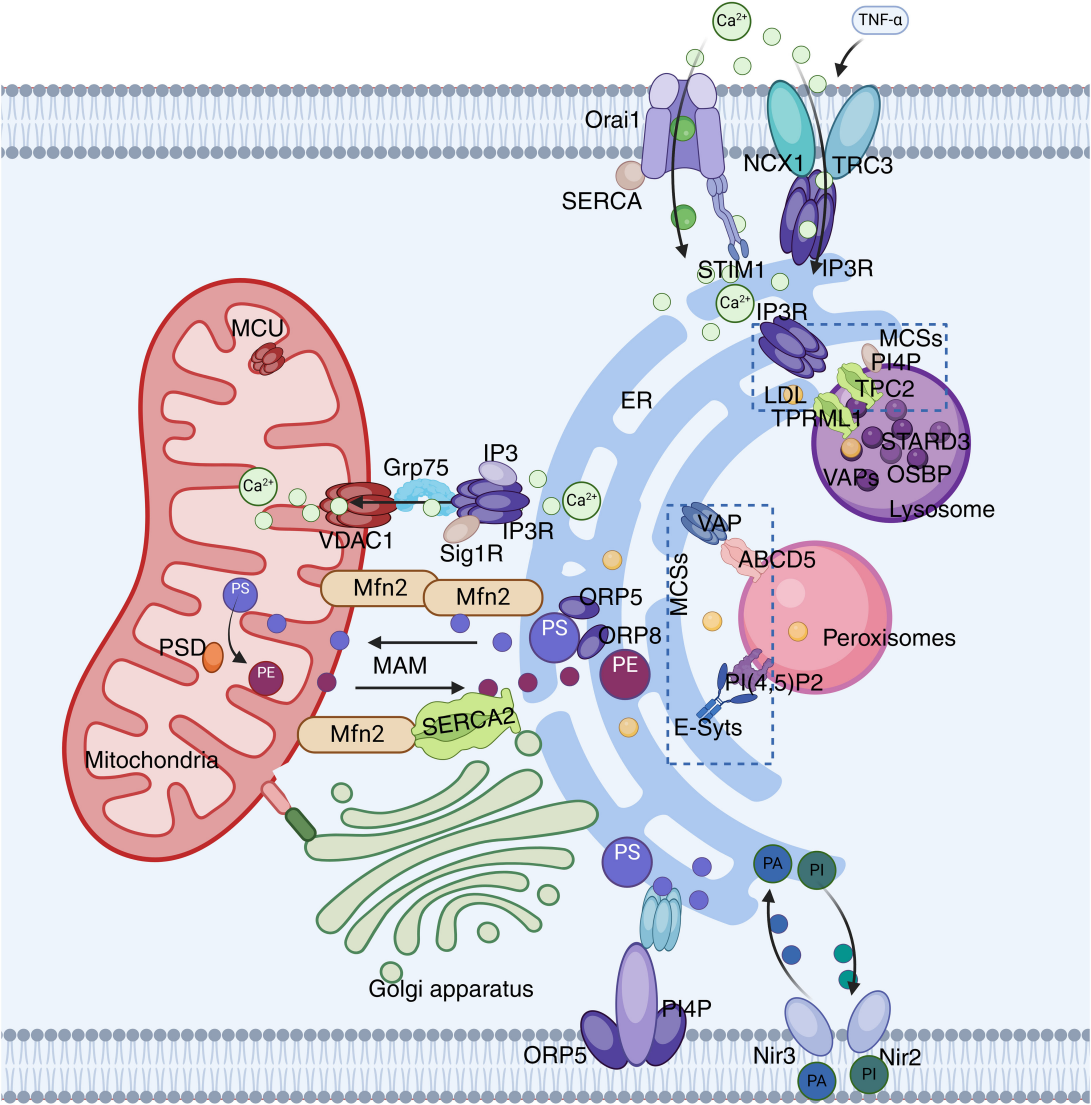
ER network. The ER interacts with mitochondria through the IP3R-GRP75-VDAC-MCU complex. VAPs and OSBP play important roles in the interactions between the ER and the Golgi apparatus or endosomes. VAP also plays a significant role in ER-peroxisome interactions by contacting ACBD5. FATP1 and DGAT2 bridge the ER to LD. The ER is also anchored to the PM through STIM1-Orai1 interactions.

The ER, which serves as the starting point of the membrane system, communicates with almost all membrane-bound organelles (23, 24), such as the mitochondria, LD, Golgi apparatus, lysosomes, and the plasma membrane (PM). In addition, the ER is also connected to membraneless organelles (24, 25), thus forming a hub of the intracellular network (**Figure 1**).

### 3.1 ER-mitochondria interactions

Microscopic observations reveal that the ER and mitochondria are connected through multiple contact sites to form specific regions known as the mitochondria associated membrane (MAM) (26). The MAM is a highly variable and dynamic structure, and its maintenance of normal morphology during dynamic processes depends on certain protein molecules that serve as physical connections, such as the calcium ion channel inositol 1,4,5-trisphosphate receptor(IP3R), molecular chaperone glucose-regulated protein 75 (Grp75), ER-mitochondria encounter structure (ERMES), and mitochondrial fusion protein 2 (MFN2) (27). Previous studies (28, 29) have found that the ER and mitochondria interact extensively and frequently through MAMs, not only playing a crucial role in maintaining the functions of the ER and mitochondria but also becoming a core hub for integrating intracellular Ca^2+^ signaling and regulating lipid metabolism.

#### 3.1.1 ER-mitochondrial interactions and Ca^2+^ transport

The ER is the primary calcium storage organelle within the cell, and the accumulation of Ca^2+^ within mitochondria largely depends on it. IP3R is a Ca^2+^ release channel on the ER that is activated by IP3, and it controls the transfer of Ca^2+^ from the ER to mitochondria through the MAM (30). In contrast, the voltage dependent anion channel 1 (VDAC1) interacts with IP3R through GRP75 on the MAM, forming an IP3R1-GRP75-VDAC1 complex, which directly facilitates the transfer of Ca^2+^ from the ER to mitochondria (31). Additionally, there are other proteins related to Ca^2+^ transfer, such as the Sigma-1 receptor (Sig1R), which is enriched at the MAM and regulates the exchange of Ca^2+^ between the ER and mitochondria by interacting with IP3R (32).

In addition to the aforementioned ion channels, MAMs possess a variety of Ca^2+^ regulatory proteins. MFN2 can promote the influx of Ca^2+^ into mitochondria by increasing the surface area of MAMs. MFN2 can also stimulate the sarcoplasmic/ER Ca^2+^-ATPase 2a (SERCA2) on MAMs, preventing excessive Ca^2+^ accumulation in mitochondria and apoptosis by regulating Ca^2+^ uptake (33). Furthermore, MAMs regulate the Ca^2+^ concentration through various pathways, maintaining it at a normal level as far as possible, which is of great significance for cellular homeostasis.

#### 3.1.2 ER-mitochondrial interactions and lipid transfer

In addition to regulating Ca^2+^ transfer, another important function of MAMs is the exchange of phospholipids between organelles. Since mitochondria cannot synthesize phosphatidylserine (PS), they must import it from the ER. PS is transferred to the mitochondria through MAMs and then converted into phosphatidylethanolamine (PE) by phosphatidylserine decarboxylase (PSD), with PE eventually returning to the ER (34). Furthermore, MFN2 and the ER proteins ORP5/8 can specifically bind to PS, regulating its transfer to the mitochondria to be converted to PE (35).

#### 3.1.3 ER-mitochondrial interactions and mitochondrial dynamics

A more detailed description is provided in Section 4.1.

### 3.2 ER and PM interactions

Electron microscopy reveals close contact between the ER and PM. This contact is not merely membrane fusion but involves interactions between specific proteins on the ER membrane and the plasma membrane (36). Known connection sites on the plasma membrane include transient receptor potential canonical (TRPC), Na^+^/Ca^2+^ exchangers (NCX), as well as proteins like junctin and Nir2 that directly span the ER and PM (37). Many important physiological activities occur at these connection sites, such as lipid transport and the maintenance of Ca^2+^ homeostasis.

#### 3.2.1 ER-PM interaction and Ca^2+^ transport

The PM is an important barrier that prevents extracellular Ca^2+^ from entering the cell, and the ER-PM contact plays a crucial role in the transfer of Ca^2+^ signals. The stromal interaction molecule (the STIM protein) dynamically regulates Ca^2+^ signals within the cell. When the ER releases Ca^2+^, the STIM protein is activated and rapidly moves to the ER-PM contact area. At this point, the STIM protein activates the Orai channel, and subsequently, Orai1 guides the influx of extracellular Ca^2+^ into the cell, activates the SERCA pump, and promotes the entry of Ca^2+^ into the ER lumen (38, 39). In this process, SOCE is activated, and TRPC1 is identified as one of its molecular components. TRPC1 can bind with Orai1 to form a TRPC-Orai1 complex to regulate Ca^2+^ signals (40). In addition to the aforementioned proteins, there are also proteins like NCX at the ER-PM contact site. It forms an NCX1-TRPC3-IP3R1 complex with TRPC3 and IP3R1, which is involved in TNF-α-induced Ca^2+^ imbalance and apoptosis (41).

#### 3.2.2 ER-PM interactions and lipid transport

ER-PM interactions play a crucial role in lipid transport. In the PM, oxysterol binding protein (OSBP)-related protein 5 (ORP5) and ORP8 bind to phosphatidylinositol-4-phosphate (PI(4)P diC 4, PI4P). They regulate the concentration of PI4P within the membrane by interacting with the Sac1 phosphatase in the ER, and can selectively enrich PS in the plasma membrane (42, 43). The binding of Nir2 and Nir3 to the PM involves the metabolite PA of PI (4,5) P2. They are responsible for transporting PA from the plasma membrane to the ER and transporting phosphatidylinositol (PI) from the ER to the PM, thereby providing the necessary components for the production of PI (4,5) P2 in the PM (43).

### 3.3 Interaction between the ER and lysosomes

#### 3.3.1 ER-lysosome interaction and Ca^2+^ transport

The interaction between the ER and lysosomes plays a crucial role in cell biology, particularly in regulating intracellular Ca^2+^ signals [ (44). The interaction between the ER and lysosomes facilitates Ca^2+^ transport through the formation of MCSs (45, 46). Studies have found that the IP3 receptors (IP3R) in the ER and the transient receptor potential channels (TPRML1 and TPC2) in lysosomes are involved in this process, regulating the release and influx of Ca^2+^ (47). In addition, the regulation of Ca^2+^ signaling by lysosomes is also considered an important factor in controlling cell death and survival (48, 49).

#### 3.3.2 ER-lysosome interactions and lipid transport

Lysosomes play a crucial role in cellular processes such as endocytosis and exocytosis. Endocytic products are initially found in early endosomes (EEs) and gradually mature into late endosomes (LEs). From these compartments, cargos can be sorted to different destinations—either returning to the PM or being transported to lysosomes via intraluminal vesicles (50). Recently, extensive MCSs between the ER and endosomes have been observed in many cell types (51). Interactions between the ER and lysosomes are important for intracellular lipid transport. As endosomes mature, the frequency of contact between them and the ER significantly increases, with this phenomenon occurring in about 50% of EEs and over 99% of LEs (52, 53). Cholesterol within endosomes is primarily acquired through the endocytosis of low-density lipoproteins, after which it is transported to downstream organelles, including the PM, ER, and mitochondria, during the endosome transport process to perform its physiological functions (54). Approximately 30% of endosomal cholesterol is transferred to the ER via MCSs, the process involving the interaction of multiple proteins, such as VAPs and endosome-localized proteins STARD3, ORP1L, and OSBP, which play key roles in maintaining ER-endosome MCSs and regulating lipid transport (55, 56). Furthermore, the interaction between VAPs and ORP1L promotes the transport of cholesterol from LEs to the ER, while STARD3 acts in the contrary effect by binding and transferring cholesterol between membranes (57, 58). The interaction between VAP and OSBP facilitates phospholipid homeostasis in ER-endosome MCSs by recruiting the PI(4)P phosphatase Sac1 to the MCS, further reducing the level of PI(4)P in endosomes (59).

### 3.4 ER-peroxisome interactions

Peroxisomes, which arise from the division of either existing peroxisomes or the ER, are involved in important functions such as lipid synthesis, fatty acid breakdown, and detoxification (5, 60, 61). They interact with other organelles through vesicular transport or MCSs, with interactions with the ER being particularly crucial for the biosynthesis of sterols and unsaturated fatty acids (62). Peroxisomes lack enzymes for autonomous lipid synthesis, and the required phospholipids are provided by the ER, with lipid transfer depending on the MCS between the ER and peroxisomes (36, 63). Studies have shown that ACBD5 in peroxisomes interacts with VAPs in the ER, and the resulting MCS plays an important role in lipid transfer and organelle maintenance (64). VAPs or ACBD5 overexpression can increase the number and surface area of MCSs (36). Additionally, PI(4,5)P2 in peroxisomes interacts with E-Syts in the ER, promoting cholesterol transfer. The MCS between the ER and peroxisomes also plays a significant role in the migration and distribution of peroxisomes, and its disruption can increase the quantity of movements and displacement of peroxisomes (65).

## 4 The mitochondrial network

**Figure 2:**
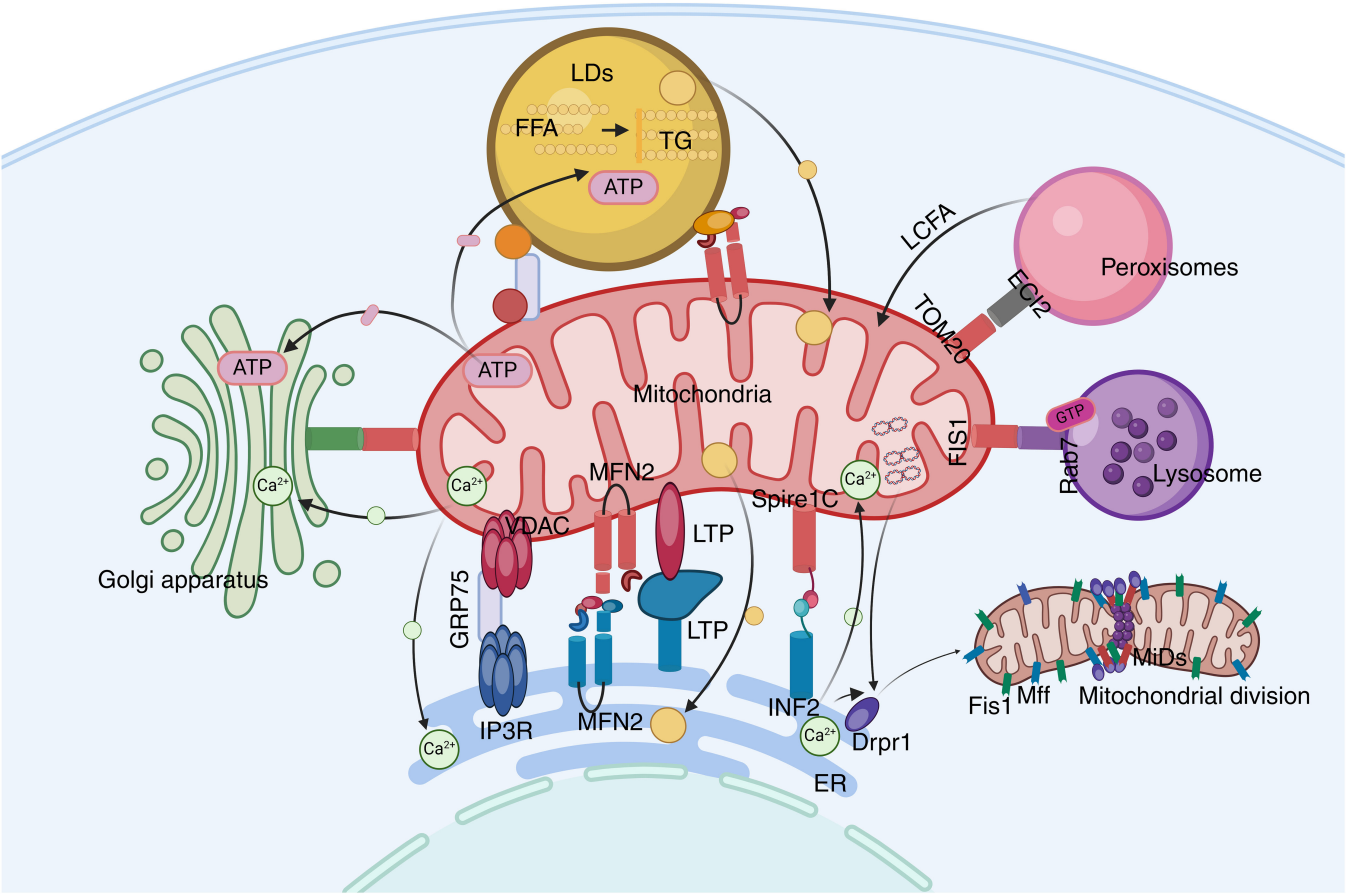
The mitochondrial network. Mitochondria play a central role in cellular metabolism and interact with other organelles. ECI2/ACBD2 plays an important role in the mitochondrial-peroxisome MCS. Rab7 is crucial in mitochondrial-lysosome interactions. LDs can recruit mitochondria through DGAT2 and perilipin5.

Mitochondria play a central role in cellular metabolism, serving as the site where ATP is synthesized through processes such as oxidative phosphorylation and the tricarboxylic acid (TCA) cycle (66). The interactions between mitochondria and other organelles, such as the ER, lysosomes, and peroxisomes, form a complex network. These interactions are facilitated through vesicle transport and MCSs, ensuring the effective transfer of materials and signals within the cell (67, 68) (**Figure 2**).

### 4.1 ER-mitochondria interactions and mitochondrial dynamics

Mitochondrial dynamics refers to the process by which mitochondria undergo cycles of fusion and fission, altering their shape, size, and location. ER-mitochondria interactions closely regulate the fission and fusion of mitochondria.

#### 4.1.1 ER-mitochondrial interactions and mitochondrial fission

Mitochondrial fission refers to the process by which mitochondria are gradually divided into smaller ring-like structures within the cell. Key molecules involved in mitochondrial fission include mitochondrial dynamin 1 (Drp1), mitochondrial dynamin 2 (Dnm2), mitochondrial fission factor (MFF), and mitochondrial dynamin-like GTPase (MiD49 and MiD51) (69). Mitochondrial fission begins with the replication of mtDNA. ER markers recruit sites where Drp1 assembles into oligomers at the pre-constriction sites marked by the ER. At the mitochondrial-ER contact sites, the ER binds to mitochondrial fusion protein 2 (MFN2) and mitochondrial Spire1C, inducing actin nucleation and polymerization. Myosin II A at the contact sites provides mechanical force to drive mitochondrial pre-constriction. At these sites, MFF and MiDs recruit Drp1, which oligomerizes into a ring-like structure. GTP hydrolysis enhances membrane constriction, and then Dnm2 is recruited to the constriction site for assembly and membrane scission, resulting in the formation of two mitochondrial progenies (70).

Additionally, mitochondrial fission requires sufficient Ca^2+^ to complete organelle constriction. INF2 can promote the transport of Ca^2+^ from the ER to the mitochondria by enhancing the contact strength between organelle membranes at MAMs, aiding in the completion of mitochondrial fission (71). Numerous studies have demonstrated that Golgi-derived vesicles containing PI(4)P are recruited to mitochondrial-ER MCSs and can activate DRP1-mediated mitochondrial fission. The disruption of PI(4)P production leads to the prevention of mitochondrial fission and mitochondrial morphology defects (72). Mitochondrial lysosomal MCSs also play a significant role in mitochondrial fission. Studies indicate that over 80% of mitochondrial fission events are marked by lysosomal marker lysosome-associated membrane protein 1 (LAMP1)-positive vesicles (21, 73, 74).

#### 4.1.2 ER-mitochondrial interactions and mitochondrial fusion

Mitochondrial fusion refers to the process whereby two or more mitochondria merge within a cell to form a larger mitochondrion. This process requires the activation of GTPases associated with actin, such as the mitochondrial fusion proteins 1 and 2 (Mfn1/2) on the outer mitochondrial membrane (OMM), and the optic atrophy protein 1 (Opa1) on the inner mitochondrial membrane (IMM), which sequentially fuse the OMM, IMM, and the internal components of the mitochondria (75). During the fusion of the outer mitochondrial membrane, Mfn1 and Mfn2 on the OMMs of two adjacent mitochondria connect with each other to initiate the fusion of the OMM (70, 76). After the fusion of the OMM, Opa1 and cardiolipin (CL) mediate the fusion of the IMM. Following GTP hydrolysis, the interaction between Opa1 and cardiolipin (CL) on both sides of the membrane bundles the two IMMs together, completing the fusion of the IMM (77).

### 4.2 Mitochondria and PM interaction

It has been demonstrated under the microscope that there is an interaction between mitochondria and the PM (78). Mitochondria located beneath the plasma membrane transfer Ca^2+^ to the ER) by regulating the activity of Ca^2+^-ATPase in the PM (79). In addition, the PM-mitochondrial MCS also play a role in the distribution of mitochondria during cell division (80, 81).

### 4.3 Mitochondria-lysosome interactions

Mitochondria and lysosomes are key organelles involved in cellular homeostasis, and they also interact with each other. Functionally, mitochondrial respiratory dysfunction can lead to lysosomal defects. For example, the absence of mitochondrial proteins AIF, OPAI, or PINK1, which affects mitochondrial function, can impair lysosomal activity (82). Conversely, lysosomal function has been certificated to be necessary for maintaining mitochondrial homeostasis. For instance, mTORC1 in neurons promotes the mitochondrial stress response, regulates its activity, and increases the fusion of lysosomes with mitochondria; disrupting lysosomal acidification reduces mitochondrial respiration (83). Additionally, mitochondria and lysosomes can directly interact under stress conditions through mitochondrial-derived vesicles (MDVs) and mitophagy (73).

Mitochondria-lysosome MCSs are abundant. Unlike MDVs that fuse with lysosomes or mitochondria during autophagy, mitochondria-lysosome MCSs do not lead to substantial transfer of mitochondrial matrix contents or mitochondrial degradation (21). The average distance between membranes in mitochondria-lysosome MCSs is 10 nm, and they play a crucial role in regulating the dynamic properties of the two organelles and ensuring Ca^2+^ transfer. Rab7 is a GTPase, a key protein that can regulate the dynamic process of tethering and detaching of mitochondria-lysosome MCSs (73). Rab7 is in an active GTP-bound state under normal conditions; however, it becomes inactive in a GDP-bound state when GTP is hydrolyzed (21). The interaction between GTP-Rab7 in lysosomes and Rab7 effectors in mitochondria plays a central role in maintaining the stability of mitochondria-lysosome MCSs. However, the mitochondrial outer membrane (OMM) protein FIS1 recruits the cytoplasmic TBC domain family member 15 (TBC1D15) to promote the hydrolysis of GTP-bound Rab7 to a GDP-bound state, thereby leading to the dissociation of MCSs (73).

### 4.4 Mitochondria and peroxisomes interaction

Both mitochondria and peroxisomes play important roles in lipid and reactive oxygen species (ROS) metabolism (84). Studies have found that the mitochondrial outer membrane translocase TOM20 and the peroxisomal enoyl-CoA isomerase ECI2 interact with peroxisomes (84). Peroxisomes play a crucial role in the oxidation of long-chain fatty acids, which are then transferred to mitochondria for complete β-oxidation, suggesting that the interaction between mitochondria and peroxisomes plays an important role in the regulation of lipid metabolism (85).

### 4.5 Mitochondria and lipid droplet interactions

A more detailed description is provided in section 5.2.

## 5 Lipid droplet network system

**Figure 3:**
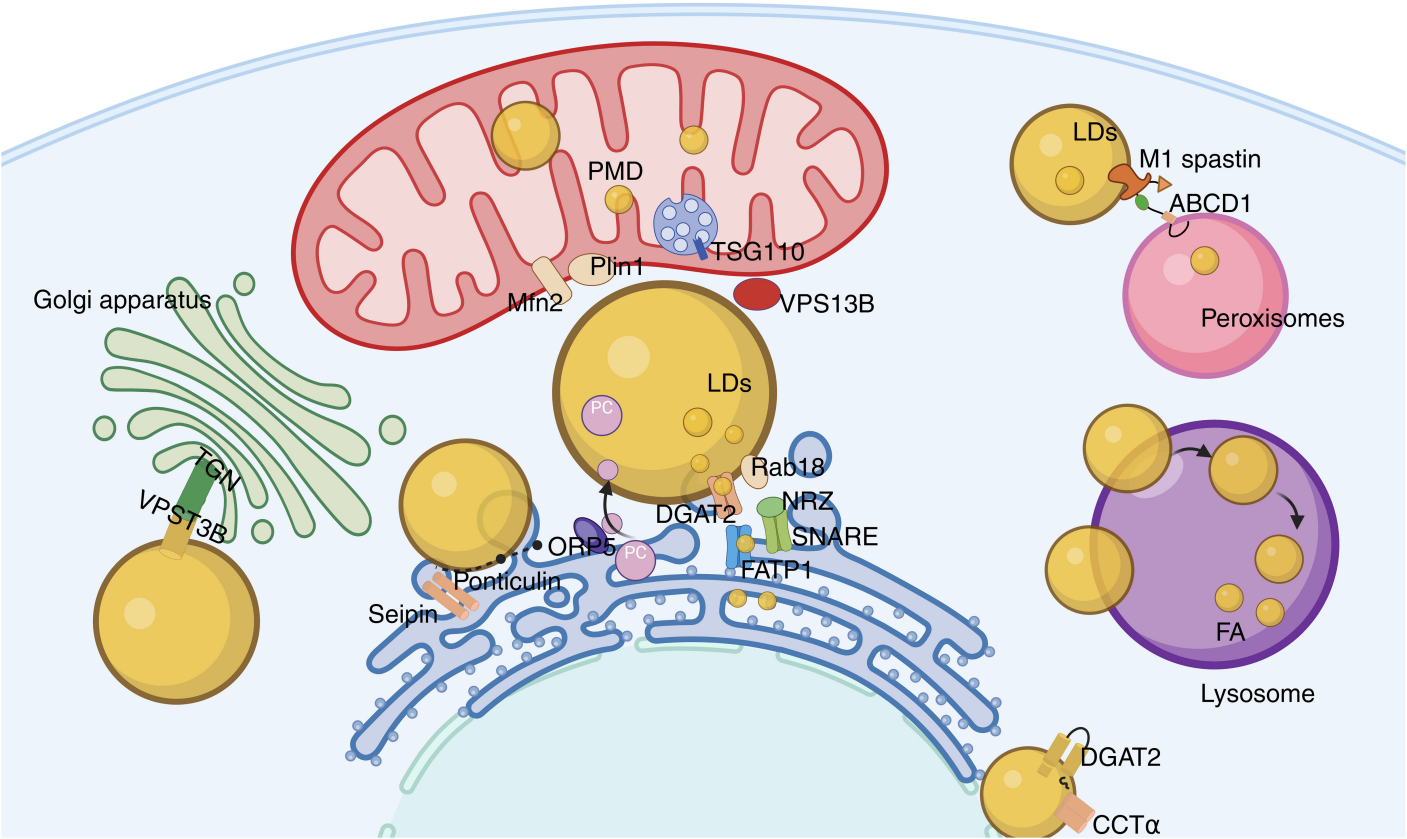
Lipid droplet network system. The contact between LD and ER occurs through membrane bridges and classical MCS, with Rab18, FATP1, and DGAT2 playing key roles in triglyceride synthesis and lipid droplet expansion; the binding of LD to mitochondria promotes the exchange of energy and metabolites, and the interaction between Mfn2 and Plin1 enhances this interaction; VPS13B maintains the integrity of the Golgi apparatus, affecting its secretory function; the contact between LD and peroxisomes promotes fatty acid metabolism and prevents lipotoxic damage.

LDs are multifunctional membrane-bound organelles with a neutral lipid core that are capable of interacting with various other organelles (86–88), playing a significant role in metabolic regulation (**Figure 3**).

### 5.1 LD-ER interactions

LDs originate from the ER (89). Interactions between ERs are mainly of two types. One type is the membrane bridge, which is composed of continuous ER and LD membranes, and the Seipin protein is a key component that promotes the stable binding of the membrane bridge (90). Mutations in Seipin can lead to abnormalities in the contact interface between ER and LD, indicating that it may play a role in stabilizing contact points during the formation of the membrane bridge (91, 92). The second type is the classical membrane contact site (MCS). In ER-LD membrane contact sites, the ER-located protein ORP5 plays an important role in the transfer of PC from ER to LD (93). The tethering proteins of ER-LD MCS are composed of various components, including the ER-located fatty acid transport protein 1 (FATP1), the LD-located diacylglycerol acyltransferase 2 (DGAT2), as well as the ER-associated NAG-RINT1-ZW10 (NRZ) complex and related SNAREs (Syntaxin-18, Use1, BNIP1), and also include the LD-located Rab18 (36, 94, 95). Rab18 is associated with lipolysis and lipogenesis, and its overexpression causes the ER and LDs to be closely adjacent. Studies have demonstrated that the interaction of Rab18-NRZ-SNARE regulates the synthesis of triglycerides in the ER, thereby promoting the growth and maturation of LDs (94, 95). In addition, the FATP1 and DGAT2 complex combines the synthesis of triglycerides with LD deposition to maintain ER-LD membrane contact sites and promote LD expansion (94, 96).

### 5.2 Interaction between LDs and mitochondria

The contact between LDs and mitochondria promotes the efficient exchange of energy and metabolites. In brown adipocytes, mitochondria exist around LDs, and such mitochondria are referred to as peri-droplet mitochondria (PDM) (97). Further studies have shown that PDMs are firmly attached to LDs and remain stable. Even after management with detergents or trypsin, the connection between them remains intact. PDMs exhibit strong capabilities in pyruvate oxidation and ATP synthesis, while their ability for fatty acid β-oxidation is relatively weak. These functional characteristics not only provide ATP for the synthesis of triglycerides (TG) but also promote the storage of fatty acids in the form of TGs in LDs, reducing fatty acid oxidation (98). Additionally, adrenergic stimulation can induce the binding of mitochondrial surface protein Mfn2 with Plin1. This binding mediates the interaction between LDs and mitochondria (99). Proteins of the ESCRT family, TSG101 and VPS13D (vacuolar protein sorting-associated protein 13D), can mediate the interaction between LDs and mitochondria. This enhances the mitochondrion’s acquisition and utilization of neutral lipids from LDs (100).

### 5.3 Interaction between LD and the Golgi apparatus

The interaction between LDs and the Golgi apparatus may affect the secretory function of the latter. The trans-Golgi network (TGN), located at specific sites, is an important sorting structure for proteins and lipids within the cell. VPS13B can form membrane contact sites between the TGN and LD, thereby maintaining the integrity and function of the TGN. VPS13B dysfunction can lead to protein secretion disorders and lipid metabolism imbalance (86).

### 5.4 LDs interact with peroxisomes

Research indicates that 10% of LDs make contact with peroxisomes (63). The peroxisome-LD MCS may link lactate dehydrogenase-mediated lipolysis with the fatty acid β-oxidation process within peroxisomes. The LD protein M1 spastin interacts with the peroxisomal protein ATP-binding cassette subfamily D member 1 (ABCD1), promoting the formation of MCS between peroxisomes and LDs, thereby assisting fatty acids in crossing the organelle boundary and preventing lipotoxic damage (101, 102).

## 6 The role of organelle interactions in NAFLD

### 6.1 Dysfunction of membrane contact sites and NAFLD

An increasing number of studies have indicated that the disruption of organelle interaction networks is closely related to the development of various diseases. Among these studies, the interaction between the ER and mitochondria has attracted particular attention, with the most in-depth research conducted in the context of NAFLD. In the livers of patients with NAFLD, the integrity of the MAM is compromised. This damage manifests as changes in the spatial distance between membranes and alterations in the protein composition of the MAM. For instance, in the livers of NAFLD mice induced by a high-fat diet, the levels of MAM-associated proteins such as IP3R1/2, MFN2, Sig-1R, and PACS-2 are significantly elevated (103). The number of MAMs in patients with NAFLD is significantly increased and correlates positively with the degree of fatty liver (104). Furthermore, overactivated MAMs trigger mitochondrial calcium overload, leading to excessive ROS production, oxidative stress (105), the opening of the mitochondrial permeability transition pore, and other mitochondrial dysfunctions, which, in turn, cause cell apoptosis (106), cellular senescence (107), metabolic disorders, insulin resistance, and cellular steatosis (108).

#### 6.1.1 The impact of MAMs on mitochondrial calcium overload and NAFLD

A more detailed description is provided in Section 6.3.

#### 6.1.2 MAMs regulate lipid synthesis and transport

A more detailed description is provided in Section 6.2.

#### 6.1.3 The impact of MAMs on mitochondrial autophagy and NAFLD

Autophagy, a self-digestion process that occurs within eukaryotic cells, is highly evolutionarily conserved. Its characteristic feature is the formation of autophagosomes, which are double-membrane vesicles with a membrane structure derived from the ER. Cells maintain homeostasis by clearing dysfunctional or damaged mitochondria through a process known as “mitochondrial autophagy.” Existing evidence suggests that core proteins play a role in MAMs, participating in PINK1/Parkin-mediated mitochondrial autophagy and regulating the integrity and function of MAMs (109). Beclin1 is located at MAMs, it promotes the increase the number of MAMs and the formation of autophagosome precursors together with PINK1 (110). Studies have found that in diabetic model mice, the expression levels of Parkin and PINK1 are reduced, which in turn decreases the number of mitochondrial autophagosomes and increases oxidative stress in liver cells (111); similarly, in the livers of NAFLD model rats, PINK1-Parkin-mediated mitochondrial autophagy is significantly reduced, leading to the accumulation of damaged mitochondria (112). Activating PINK1-Parkin-mediated mitochondrial autophagy can improve fat deposition and mitochondrial damage in liver cells of patients with NAFLD (113–115). In addition, the mitochondrial autophagy-related protein FUNDC1 accumulates in MAMs by interacting with IP3 receptor 2 in MAMs and promotes the formation of MAMs and the release of Ca^2+^ from the ER to mitochondria (116, 117). Mice lacking FUNDC1 exhibit severe obesity and insulin resistance (118). In summary, mitochondrial autophagy is a key mechanism in the pathogenesis of NAFLD, and the structure and function of MAMs are crucial to this mechanism. Therefore, regulating MAMs to affect mitochondrial autophagy may become a new target for the treatment of NAFLD.

#### 6.1.4 The impact of MAMs on mitochondrial ROS generation and NAFLD

At present, several Ca^2+^ channel regulators have been identified in MAMs. These regulators can modulate the production of mitochondrial reactive oxygen species (mtROS). At the molecular level, ERo1α oxidizes IP3R1, prompting the dissociation of ERp44 from IP3R1, thereby enhancing the transfer of Ca^2+^ from the ER to the mitochondria, leading to excessive mtROS generation. Conversely, through ERo1α-dependent mechanisms, Ca^2+^ signaling at MAMs is enhanced (119). In addition, MAMs can also directly generate mtROS through P66Sh (120). Under normal conditions, ROS maintain cellular homeostasis and play a role in signal transduction. When mitochondria are damaged, ROS levels increase. ROS accumulation in hepatocytes affects insulin and inflammatory signaling, promoting the occurrence of insulin resistance and inflammation, thereby further advancing NAFLD (121). Studies have shown that activating the AMPK signaling pathway can promote mitochondrial biogenesis and energy metabolism in the livers of HFD-induced NAFLD mice, reduce cellular ROS levels, and thereby alleviate hepatic lipid deposition (122).

#### 6.1.5 The impact of MAMs on cellular senescence and NAFLD

Cellular senescence is a state induced by stress signals that cause cells to enter a cell cycle arrest and lose their ability to proliferate (123). Studies have shown that the levels of senescence-associated markers p16, p21, and SA-β-Gal in fatty liver are positively correlated with the grade of NAFLD, insulin resistance, hepatic inflammation, and fibrosis levels (124). Recent research indicates that MAMs play a significant role in cellular senescence and are deeply involved in the pathogenesis of NAFLD (125). The number of MAMs in the livers of HFD mice and ob/ob mice is significantly increased. This change is closely related to hepatic insulin resistance and steatosis (103). Introducing exogenous MAM connectors into normal mice can promote MAM formation. This process can induce the occurrence of insulin resistance. Conversely, knocking down IP3R2 to inhibit MAMs formation can significantly improve glucose homeostasis, alleviate cellular senescence, and thus effectively alleviate hepatic steatosis (107).

#### 6.1.6 The impact of MAMs on unfolded protein response and NAFLD

Under stress conditions, cells accumulate unfolded or misfolded proteins, leading to ER stress (ERS). To maintain the function of the ER and the homeostasis of intracellular proteins, the UPR is activated by transmembrane stress sensors located in the ER, which include inositol-requiring enzyme 1α (IRE1α), RNA-dependent protein kinase-like ER kinase (PERK), and transcription factor 6α (ATF6α) (126). Notably, UPR proteins IRE1α and PERK are distributed on MAMs. The MAM-located E3 ubiquitin ligase MITOL inhibits the overactivation of IRE1α by ubiquitinating it. When MITOL is absent, apoptosis increases. Additionally, reducing PACS2 expression can decrease the interaction between MITOL and IRE1α and also lower the ubiquitination level of IRE1α. Moreover, MAMs play a role in the attenuation of IRE1α signaling after ERS is resolved (127).

Although the absence of MFN2 enhances UPR activation, it also mitigates the degree of apoptosis, and inhibiting PERK can partially reverse the phenotype caused by MFN2 deficiency (128). MFN2 directly interacts with PERK and may act as an enhancer regulator of PERK, while the absence of MFN2 may lead to ERS (129). Studies have found that a transient UPR can inhibit steatosis, while sustained high levels of ERS can exacerbate hepatic steatosis, ultimately leading to UPR-induced apoptosis and promoting the occurrence and progression of NAFLD. In patients with NAFLD, UPR initiation activates the c-Jun N-terminal kinase (JNK) signaling pathway, leading to inflammation and apoptosis (130). Under high-fat diet conditions, the loss of JNK function can protect mice from insulin resistance. Furthermore, the UPR pathway can directly or indirectly regulate the expression of lipid synthesis enzymes, thereby increasing lipid synthesis and storage. UPR activates the key transcription factor in lipid metabolism—the liver X receptor 1c. Its overactivation leads to hepatic fat deposition and further exacerbates ERS (131).

### 6.2 Homeostasis and NAFLD

LD plays a coordinating role in lipid synthesis, storage, secretion, and degradation, which are crucial for maintaining lipid balance (132). LD accumulation in hepatocytes leads to steatosis, which is the main pathological feature of NAFLD, and the interactions between LDs and organelles have become a new focus of NAFLD research.

#### 6.2.1 LDs and ER in NAFLD

The liver’s fat mainly comes from three sources: free fatty acids (FFA) produced by the breakdown of adipose tissue, *de novo* fatty acid synthesis, and dietary intake. The liver synthesizes fat primarily in the form of very low-density lipoprotein (VLDL). An increase in fatty acid sources and impaired fat secretion are the main reasons for the increased lipid levels in the livers of patients with NAFLD (133). The interaction between LDs and ER plays an important role in the processes of fat breakdown and VLDL synthesis and secretion (134). In hepatocytes, apolipoprotein B (ApoB) is a key molecule for VLDL assembly and secretion. At the same time, the interaction between LDs and ER affects VLDL assembly by regulating ApoB degradation. After ApoB binds to lipids on the ER luminal side, it accumulates on LD. Subsequently, ApoB is transferred from the ER luminal side to the cytoplasmic side, binds to UBXD8 on LDs after ubiquitination, and is transported to the proteasome for degradation. In mice with UBXD8 gene deletion, the VLDL level in the serum is significantly reduced, and the degree of hepatic steatosis is also significantly increased (134). In the liver tissue of mice with NAFLD fed with a high-fat diet, the contact between LDs and ER increases may affect the storage and output of fat (135).

#### 6.2.2 LD and mitochondria in NAFLD

Researchers have observed via electron microscopy that in the livers of mice fed with a high-fat diet, mitochondria are in contact with LDs (103). Studies have found that Plin5 can promote the contact between mitochondria and LDs to form PDMs (98, 136). Plin5 can also facilitate the contact between mitochondria and LDs, form LD-mitochondria contact sites, and store fatty acids as TGs, help to buffer excess FFAs, thereby protecting hepatocytes from lipotoxic effects (137). In addition, Plin5, by promoting contact between LDs and mitochondria, can upregulate genes related to mitochondrial function, reduce ROS levels in cells, thus, protect hepatocytes from oxidative stress damage (138). If Plin5 is knocked out, hepatic steatosis is alleviated, but the free FFAs produced by lipolysis increase, leading to lipotoxic damage (139). On the other hand, when the content of fatty acids in the cytoplasm increases, the contact protein Plin5 between mitochondria and LDs also increases, prompting fatty acids to directly enter mitochondria from LDs, enhancing the oxidation and utilization of fatty acids, and reducing the toxicity brought by high concentrations of fatty acids in the cytoplasm (140, 141).

#### 6.2.3 Interaction of LD with other organelles and NAFLD

Lysosomes interact with LDs to form autophagosomes. Autophagosomes break down neutral lipids within LDs, release FFAs, which are then used for energy metabolism. This process is known as lipophagy. In the liver, lipophagy is the main pathway for lipid breakdown, and in the liver tissue of patients with NAFLD, this process often occurs abnormally (142). Oxidative stress and lipid peroxidation are closely related to the occurrence of NAFLD, while antioxidant enzymes and enzymes that produce ROS in peroxisomes play an important role in maintaining the balance of fatty acid β-oxidation and redox homeostasis. In cells treated with oleic acid, peroxisomes form stable membrane contact sites with LDs, and their inner membranes can extend into the interior of LDs (86). Proteomic analysis of extracted lipid droplet LDs revealed significant enrichment of peroxisomal β-oxidation enzymes within LDs (135).

### 6.3 Calcium ion homeostasis and NAFLD

#### 6.3.1 ER and mitochondrial calcium homeostasis

Research indicates that the MAM plays a crucial role in maintaining Ca^2+^ homeostasis, and its dysfunction is a key link leading to lipid deposition in hepatocytes and mitochondrial calcium overload (143). The expression levels of Ca^2+^ channel proteins associated with MAM are positively correlated with the concentration of MAM (144). In the livers of obese mice, the fat deposition in hepatocytes increases the number of contact points between the ER and mitochondria and also enhances the transfer of Ca^2+^ from the ER to the mitochondria (145). This leads to mitochondrial calcium overload, which in turn triggers a series of adverse consequences such as mitochondrial dysfunction, increased ROS production, weakened insulin action in the liver, and metabolic abnormalities, further exacerbated the deposition of liver lipids and formed a vicious cycle (103). Studies have demonstrated that during the development of NAFLD, the activity of SERCA within hepatocytes significantly decreases, leading to reduced Ca^2+^ input and increased output in the ER, thereby causing mitochondrial calcium overload and ERS (146). Restoring SERCA activity significantly reduces the levels of ERS-related markers, such as glucose-regulated protein 78, sterol regulatory element-binding protein, phosphorylated PERK, phosphorylated eIF2α, and C/EBP homologous protein, while also alleviating lipid deposition in hepatocytes (146, 147). In addition, CDGSH iron-sulfur domain-containing protein 2 can regulate the oxidative modification of SERCA2b, increase ER Ca^2+^ uptake, maintain Ca^2+^ homeostasis, and inhibit mitochondrial calcium overload and ERS, thereby improving NAFLD (148). Furthermore, downregulating the levels of MAM-associated proteins IP3R1 and PACS-2 can improve lipid deposition in hepatocytes caused by Ca^2+^ disorder, thereby reducing the burden on the liver (103).

#### 6.3.2 Lysosomal calcium homeostasis

Lysosomal calcium homeostasis plays a crucial role in maintaining lysosomal function. The balance of lysosomal calcium is essential for its function, and its disruption can lead to diseases associated with lysosomal acidification (149). This is related to the proteolytic activity of lysosomes (150). Furthermore, impaired proteolytic activity in lysosomes can negatively affect the autophagy proteolysis system. This impact ultimately leads to cellular senescence (151–155). In senescent cells, damaged mitochondria accumulate (156). Further research has found that dysfunction of the mitochondrial respiratory chain can inhibit the lysosomal calcium release channel MCOLN1 (also known as TRPML1), thereby disrupting lysosomal calcium homeostasis (157). The regulation of lysosomal calcium release plays an important role in controlling lysosomal biogenesis, exocytosis, and autophagy (158). Impairment of TRPML1 Function results in enlarged lysosomes, calcium ion accumulation, and increased pH, leading to lysosomal dysfunction and ultimately cellular senescence (150, 158). Studies have shown that ROS produced by mitochondria can activate TRPML1, thereby inducing lysosomal calcium release (73), further inducing autophagy and lysosomal biogenesis (159) and delaying cellular senescence. However, excessive ROS levels in mitochondria have the opposite effect, leading to lysosomal dysfunction and cellular senescence (158, 159). In summary, these data suggest that the calcium balance of lysosomes may lead to NAFLD-induced liver dysfunction.

### 6.4 Mitochondrial dynamics and NAFLD

The dynamic balance between mitochondrial fusion and fission is an essential condition for the normal functioning of cells. When this dynamic balance is disrupted, the morphology and function of mitochondria are affected. Such changes may lead to the occurrence and progression of NAFLD.

In NAFLD hepatocytes, there is an imbalance in mitochondrial dynamics, characterized by impaired mitochondrial fusion (160) and enhanced mitochondrial fission (161). This leads to alterations in the normal mitochondrial structure, disruption of the mitochondrial tubular network, fragmentation, swelling, and the appearance of short rod-like and spherical shapes. There is a decrease in the mitochondrial membrane potential, destruction of cristae structure (162), and loss of cristae (163), which inhibits cellular metabolism, affects mitochondrial function, and accelerates hepatocyte death. In comparison with normal hepatocytes, the expression levels of Mfn2 and Opa1 proteins are reduced in NAFLD hepatocytes, while the expression levels of Fis1 and DRP1 proteins are increased. Studies have found that patients with non-alcoholic steatohepatitis have significant changes in mitochondrial structure and a significant decrease in Mfn2 levels (164). By examining the expression of proteins related to mitochondrial fusion and fission in the livers of high-fat-fed mice, it was found that the expression of fission proteins (Fis1, Drp1) was enhanced, while the expression of fusion proteins (Mfn2, Opa1) decreased (165). Therefore, in NAFLD, the expression of proteins related to mitochondrial fusion decreases, while the expression of proteins related to fission increases, leading to weakened fusion, enhanced fission, and an imbalance in mitochondrial dynamics. On the other hand, increased OXPHOS activity stimulates mitochondrial fusion, while OXPHOS deficiency leads to defects in mitochondrial inner membrane fusion (166). In NAFLD, mutations in OXPHOS genes result in OXPHOS deficiency, which, in turn, leads to defects in mitochondrial inner membrane fusion, impaired mitochondrial fusion, and an imbalance in mitochondrial dynamics (167). Additionally, OXPHOS deficiency or inhibition can lead to the development of oxidative stress, increased ROS production, and further damage to the respiratory chain, preventing the complete oxidation of FFA and affecting β-oxidation (168). Therefore, the fission and fusion of mitochondria can affect fatty acid oxidation under certain circumstances (169). In summary, hepatocyte mitochondrial damage is an important mechanism in the pathogenesis of NAFLD. Abnormal mitochondrial dynamics characterized by impaired fusion and increased fission can lead to mitochondrial dysfunction and damage to hepatocytes, thereby exacerbating the pathological process of NAFLD. Correcting the imbalance in mitochondrial dynamics (promoting fusion and inhibiting fission) can effectively repair damaged mitochondria in hepatocytes and alleviate NAFLD.

## 7 Conclusion

In eukaryotic cells, fundamental biological reactions are finely compartmentalized within membrane-bound organelles. This structural separation allows incompatible biological processes to occur independently in specific microenvironments, thereby significantly enhancing cellular functional efficiency and ensure the orderly progression of various physiological activities. However, organelles do not exist in isolation; they form a complex and sophisticated biological system that requires active communication and interaction to maintain cellular homeostasis and normal function. Dysfunctional interactions between organelles can lead to a variety of intracellular issues and have profound effects on other important biological processes, such as cellular energy metabolism imbalance. Mitochondria are the primary energy producers within the cell, and their activities, particularly ATP synthesis, are finely regulated by calcium signaling to ensure that cells meet their energy demands under different physiological states. Especially at the contact points between the ER and mitochondria, calcium transfer mediated by IP3 receptors plays a crucial role in maintaining basal mitochondrial metabolism, ensuring that cells can maintain a stable supply of energy in a dynamically changing environment. Under normal cellular conditions, the inhibition of IP3 receptor activity leads to significant impairment of cellular energy metabolism, thereby affecting the overall function of the cell. In addition, peroxisomes and mitochondria are jointly involved in fatty acid degradation. Dysfunctional mitochondria can supplement the TCA cycle through the mitochondrial retrograde signaling pathway, while peroxisomes convert fatty acids to acetyl-CoA, forming a complementary metabolic network. Due to impaired fatty acid metabolism, defects in peroxisomes have a profound impact on the pathological state of mitochondria, further exacerbating cellular metabolic disorders. Therefore, these organelles work together to form an interactive network to maintain cellular homeostasis and complete a series of complex biological processes. A deeper understanding of the interrelationships between organelle interactions will help us expand our understanding of the mechanisms of complex cellular behaviors, providing new ideas for therapeutic exploration. However, the study of these interactions is not only limited to the field of NAFLD; it also involves a variety of physiological and pathological processes. Therefore, our review provides important clues and insights for a more in-depth understanding of cellular functions from a new perspective, promoting the advancement of research in related fields.

## References

[1] Helle SC Kanfer G Kolar K Lang A Michel AH Kornmann B. Organization and function of membrane contact sites. Biochim Biophys Acta. (2013) 1833:2526–41. doi: 10.1177/25152564231183898 23380708

[2] Greenberg AS Coleman RA Kraemer FB McManaman JL Obin MS Puri V. The role of lipid droplets in metabolic disease in rodents and humans. J Clin Invest. (2011) 121:2102–10. doi: 10.1172/JCI46069 PMC3104768 21633178

[3] Huby T Gautier EL. Immune cell-mediated features of non-alcoholic steatohepatitis. Nat Rev Immunol. (2022) 22:429–43. doi: 10.1038/s41577-021-00639-3 PMC8570243 34741169

[4] Younossi ZM Koenig AB Abdelatif D Fazel Y Henry L Wymer M. Global epidemiology of nonalcoholic fatty liver disease-Meta-analytic assessment of prevalence, incidence, and outcomes. Hepatology. (2016) 64:73–84. doi: 10.1002/hep.28431 26707365

[5] Cohen S Valm AM Lippincott-Schwartz J. Interacting organelles. Curr Opin Cell Biol. (2018) 53:84–91. doi: 10.1016/j 30006038 PMC6241252

[6] Stevenson J Huang EY Olzmann JA. Endoplasmic reticulum-associated degradation and lipid homeostasis. Annu Rev Nutr. (2016) 36:511–42. doi: 10.1146/annurev-nutr-071715-051030 PMC6268005 27296502

[7] Oakes SA Papa FR. The role of endoplasmic reticulum stress in human pathology. Annu Rev Pathol. (2015) 10:173–94. doi: 10.1146/annurev-pathol-012513-104649 PMC5568783 25387057

[8] Suh J Lee YS. Mitochondria as secretory organelles and therapeutic cargos. Exp Mol Med. (2024) 56:66–85. doi: 10.1038/s12276-023-01141-7 38172601 PMC10834547

[9] Madreiter-Sokolowski CT Ramadani-Muja J Ziomek G Burgstaller S Bischof H Koshenov Z. Tracking intra- and inter-organelle signaling of mitochondria. FEBS J. (2019) 286:4378–401. doi: 10.1111/febs.15103 PMC6899612 31661602

[10] Li J Ahat E Wang Y. Golgi structure and function in health, stress, and diseases. Results Probl Cell Differ. (2019) 67:441–85. doi: 10.1007/978-3-030-23173-6_19 PMC7076563 31435807

[11] Smith JJ Aitchison JD. Peroxisomes take shape. Nat Rev Mol Cell Biol. (2013) 14:803–17. doi: 10.1038/nrm3700 PMC4060825 24263361

[12] Walther TC Chung J Farese RJ. Lipid droplet biogenesis. Annu Rev Cell Dev Biol. (2017) 33:491–510. doi: 10.1146/annurev-cellbio-100616-060608 28793795 PMC6986389

[13] Toth AE Nielsen S Tomaka W Abbott NJ Nielsen MS. The endo-lysosomal system of bEnd.3 and hCMEC/D3 brain endothelial cells. Fluids Barriers CNS. (2019) 16:14. doi: 10.1186/s12987-019-0134-9 31142333 PMC6542060

[14] Lee CA Blackstone C. ER morphology and endo-lysosomal crosstalk: Functions and disease implications. Biochim Biophys Acta Mol Cell Biol Lipids. (2020) 1865:158544. doi: 10.1016/j.bbalip 31678515 PMC6904894

[15] Prinz WA Toulmay A Balla T. The functional universe of membrane contact sites. Nat Rev Mol Cell Biol. (2020) 21:7–24. doi: 10.1038/s41580-019-0180-9 31732717 PMC10619483

[16] Fernandez-Busnadiego R Saheki Y De Camilli P. Three-dimensional architecture of extended synaptotagmin-mediated endoplasmic reticulum-plasma membrane contact sites. Proc Natl Acad Sci U.S.A. (2015) 112:E2004–13. doi: 10.1073/pnas.1503191112 PMC4413308 25787254

[17] West M Zurek N Hoenger A Voeltz GK. A 3D analysis of yeast ER structure reveals how ER domains are organized by membrane curvature. J Cell Biol. (2011) 193:333–46. doi: 10.1083/jcb.201011039 PMC3080256 21502358

[18] Csordas G Weaver D Hajnoczky G. Endoplasmic reticulum-mitochondrial contactology: structure and signaling functions. Trends Cell Biol. (2018) 28:523–40. doi: 10.1016/j.tcb.2018.02.009 PMC6005738 29588129

[19] Korobova F Ramabhadran V Higgs HN. An actin-dependent step in mitochondrial fission mediated by the ER-associated formin INF2. Science. (2013) 339:464–7. doi: 10.1126/science.1228360 PMC3843506 23349293

[20] Manor U Bartholomew S Golani G Christenson E Kozlov M Higgs H. A mitochondria-anchored isoform of the actin-nucleating spire protein regulates mitochondrial division. Elife. (2015) 4:e08828. doi: 10.7554/eLife.08828 26305500 PMC4574297

[21] Wong YC Ysselstein D Krainc D. Mitochondria-lysosome contacts regulate mitochondrial fission via RAB7 GTP hydrolysis. Nature. (2018) 554:382–6. doi: 10.1038/nature25486 PMC6209448 29364868

[22] Guo Y Li D Zhang S Yang Y Liu JJ Wang X. Visualizing intracellular organelle and cytoskeletal interactions at nanoscale resolution on millisecond timescales. Cell. (2018) 175:1430–42. doi: 10.1016/j.cell.2018.09.057 30454650

[23] Phillips MJ Voeltz GK. Structure and function of ER membrane contact sites with other organelles. Nat Rev Mol Cell Biol. (2016) 17:69–82. doi: 10.1038/nrm.2015.8 26627931 PMC5117888

[24] Lee JE Cathey PI Wu H Parker R Voeltz GK. Endoplasmic reticulum contact sites regulate the dynamics of membraneless organelles. Science. (2020) 367:eaay7108. doi: 10.1126/science.aay7108 32001628 PMC10088059

[25] Lee S Bahmanyar S. The endoplasmic reticulum regulates membraneless organelles through contact sites. Biochemistry-US. (2020) 59:1716–7. doi: 10.1021/acs.biochem.0c00232 PMC10026189 32324384

[26] Fujimoto M Hayashi T. New insights into the role of mitochondria-associated endoplasmic reticulum membrane. Int Rev Cell Mol Biol. (2011) 292:73–117. doi: 10.1016/B978-0-12-386033-0.00002-5 22078959

[27] Poston CN Krishnan SC Bazemore-Walker CR. In-depth proteomic analysis of mammalian mitochondria-associated membranes (MAM). J Proteomics. (2013) 79:219–30. doi: 10.1016/j.jprot.2012.12.018 23313214

[28] Zhang P Konja D Zhang Y Wang Y. Communications between mitochondria and endoplasmic reticulum in the regulation of metabolic homeostasis. CELLS-BASEL. (2021) 10:2195. doi: 10.3390/cells10092195 PMC8468463 34571844

[29] Rowland AA Voeltz GK. Endoplasmic reticulum-mitochondria contacts: function of the junction. Nat Rev Mol Cell Biol. (2012) 13:607–25. doi: 10.1038/nrm3440 PMC5111635 22992592

[30] Ando H Kawaai K Bonneau B Mikoshiba K. Remodeling of Ca (2+) signaling in cancer: Regulation of inositol 1,4,5-trisphosphate receptors through oncogenes and tumor suppressors. Adv Biol Regul. (2018) 68:64–76. doi: 10.1016/j.jbior.2017.12.001 29287955

[31] Geng J Khaket TP Pan J Li W Zhang Y Ping Y. Deregulation of ER-mitochondria contact formation and mitochondrial calcium homeostasis mediated by VDAC in fragile X syndrome. Dev Cell. (2023) 58:597–615.e10. doi: 10.1016/j.devcel.2023.03.002 37040696 PMC10113018

[32] Honrath B Metz I Bendridi N Rieusset J Culmsee C Dolga AM. Glucose-regulated protein 75 determines ER-mitochondrial coupling and sensitivity to oxidative stress in neuronal cells. Cell Death Discovery. (2017) 3:17076. doi: 10.1038/cddiscovery.2017.76 29367884 PMC5672593

[33] Yang JF Xing X Luo L Zhou XW Feng JX Huang KB. Mitochondria-ER contact mediated by MFN2-SERCA2 interaction supports CD8(+) T cell metabolic fitness and function in tumors. Sci Immunol. (2023) 8:eabq2424. doi: 10.1126/sciimmunol.Abq2424 37738362

[34] Rieusset J. The role of endoplasmic reticulum-mitochondria contact sites in the control of glucose homeostasis: an update. Cell Death Dis. (2018) 9:388. doi: 10.1038/s41419-018-0416-1 29523782 PMC5844895

[35] Hernandez-Alvarez MI Sebastian D Vives S Ivanova S Bartoccioni P Kakimoto P. Deficient endoplasmic reticulum-mitochondrial phosphatidylserine transfer causes liver disease. Cell. (2019) 177:881–895. e17. doi: 10.1016/j.cell.2019.04.010 31051106

[36] Wu H Carvalho P Voeltz GK. Here, there, and everywhere: The importance of ER membrane contact sites. Science. (2018) 361:eaan5835. doi: 10.1126/science.aan5835 30072511 PMC6568312

[37] Kim YJ Guzman-Hernandez ML Wisniewski E Echeverria N Balla T. Phosphatidylinositol and phosphatidic acid transport between the ER and plasma membrane during PLC activation requires the Nir2 protein. Biochem Soc Trans. (2016) 44:197–201. doi: 10.1042/BST20150187 26862206 PMC6456894

[38] Zaman MF Nenadic A Radojicic A Rosado A Beh CT. Sticking with it: ER-PM membrane contact sites as a coordinating nexus for regulating lipids and proteins at the cell cortex. Front Cell Dev Biol. (2020) 8:675. doi: 10.3389/fcell.2020.00675 32793605 PMC7387695

[39] Navarro-Borelly L Somasundaram A Yamashita M Ren D Miller RJ Prakriya M. STIM1-Orai1 interactions and Orai1 conformational changes revealed by live-cell FRET microscopy. J Physiol. (2008) 586:5383–401. doi: 10.1113/jphysiol.2008 PMC2655373 18832420

[40] Maleth J Hegyi P. Calcium signaling in pancreatic ductal epithelial cells: an old friend and a nasty enemy. Cell Calcium. (2014) 55:337–45. doi: 10.1016/j.ceca.2014.02.004 24602604

[41] Chang CL Chen YJ Liou J. ER-plasma membrane junctions: Why and how do we study them? Biochim Biophys Acta Mol Cell Res. (2017) 1864:1494–506. doi: 10.1016/j.bbamcr.2017.05.018 PMC5542405 28554772

[42] Chung J Torta F Masai K Lucast L Czapla H Tanner LB. INTRACELLULAR TRANSPORT. PI4P/phosphatidylserine countertransport at ORP5- and ORP8-mediated ER-plasma membrane contacts. SCIENCE. (2015) 349:428–32. doi: 10.1126/science PMC4638224 26206935

[43] Dickson EJ Jensen JB Vivas O Kruse M Traynor-Kaplan AE Hille B. Dynamic formation of ER-PM junctions presents a lipid phosphatase to regulate phosphoinositides. J Cell Biol. (2016) 213:33–48. doi: 10.1083/jcb.201508106 27044890 PMC4828688

[44] Zhang Z Yue P Lu T Wang Y Wei Y Wei X. Role of lysosomes in physiological activities, diseases, and therapy. J Hematol Oncol. (2021) 14:79. doi: 10.1186/s13045-021-01087-1 33990205 PMC8120021

[45] Liu R Hong W Hou D Huang H Duan C. Decoding organelle interactions: unveiling molecular mechanisms and disease therapies. Adv Biol (Weinh). (2024) 8:e2300288. doi: 10.1002/adbi.202300288 38717793

[46] Shen Y Chen QC Li CY Han FJ. Independent organelle and organelle-organelle interactions: essential mechanisms for Malignant gynecological cancer cell survival. Front Immunol. (2024) 15:1393852. doi: 10.3389/fimmu.2024.1393852 38711526 PMC11070488

[47] Patel S Marchant JS Brailoiu E. Two-pore channels: Regulation by NAADP and customized roles in triggering calcium signals. Cell Calcium. (2010) 47:480–90. doi: 10.1016/j.ceca.2010.05.001 PMC2921607 20621760

[48] Zheng P Obara CJ Szczesna E Nixon-Abell J Mahalingan KK Roll-Mecak A. ER proteins decipher the tubulin code to regulate organelle distribution. Nature. (2022) 601:132–8. doi: 10.1038/s41586-021-04204-9 PMC8732269 34912111

[49] Venkatachalam K Hofmann T Montell C. Lysosomal localization of TRPML3 depends on TRPML2 and the mucolipidosis-associated protein TRPML1. J Biol Chem. (2006) 281:17517–27. doi: 10.1074/jbc.M600807200 PMC4196876 16606612

[50] Hugenroth M Bohnert M. Come a little bit closer! Lipid droplet-ER contact sites are getting crowded. Biochim Biophys Acta Mol Cell Res. (2020) 1867:118603. doi: 10.1016/j.bbamcr.2019.118603 31733263

[51] Mattiazzi UM Brloznik M Kaferle P Zitnik M Wolinski H Leitner F. Genome-wide localization study of yeast pex11 identifies peroxisome-mitochondria interactions through the ERMES complex. J Mol Biol. (2015) 427:2072–87. doi: 10.1016/j.jmb.2015.03.004 PMC4429955 25769804

[52] van der Kant R Neefjes J. Small regulators, major consequences - Ca(2)(+) and cholesterol at the endosome-ER interface. J Cell Sci. (2014) 127:929–38. doi: 10.1242/jcs.137539 24554437

[53] Raiborg C Wenzel EM Pedersen NM Stenmark H. ER-endosome contact sites in endosome positioning and protrusion outgrowth. Biochem Soc Trans. (2016) 44:441–6. doi: 10.1042/BST20150246 27068952

[54] Quinones-Frias MC. A WICB 50th Favorite: Endoplasmic reticulum-endosome contact increases as endosomes traffic and mature. Mol Biol Cell. (2021) 32:fe2. doi: 10.1091/mbc.E21-05-0240 34499529 PMC8684763

[55] Raiborg C Wenzel EM Stenmark H. ER-endosome contact sites: molecular compositions and functions. EMBO J. (2015) 34:1848–58. doi: 10.15252/embj.201591481 PMC4547891 26041457

[56] Jean S Nassari S. Regulation of endosomal sorting and maturation by ER-endosome contact sites. Contact (Thousand Oaks). (2022) 5:1214851202. doi: 10.1177/25152564221106046 PMC10243584 37366507

[57] Gong B Guo Y Ding S Liu X Meng A Li D. A Golgi-derived vesicle potentiates PtdIns4P to PtdIns3P conversion for endosome fission. Nat Cell Biol. (2021) 23:782–95. doi: 10.1038/s41556-021-00704-y 34183801

[58] Rowland AA Chitwood PJ Phillips MJ Voeltz GK. ER contact sites define the position and timing of endosome fission. Cell. (2014) 159:1027–41. doi: 10.1016/j.cell.2014.10.023 PMC4634643 25416943

[59] Eden ER Sanchez-Heras E Tsapara A Sobota A Levine TP Futter CE. Annexin A1 tethers membrane contact sites that mediate ER to endosome cholesterol transport. Dev Cell. (2016) 37:473–83. doi: 10.1016/j.devcel.2016.05.005 PMC4906250 27270042

[60] Tabak HF van der Zand A Braakman I. Peroxisomes: minted by the ER. Curr Opin Cell Biol. (2008) 20:393–400. doi: 10.1016/j.ceb.2008.05.008 18619829

[61] Joshi AS Nebenfuehr B Choudhary V Satpute-Krishnan P Levine TP Golden A. Lipid droplet and peroxisome biogenesis occur at the same ER subdomains. Nat Commun. (2018) 9:2940. doi: 10.1038/s41467-018-05277-3 30054481 PMC6063926

[62] Schuldiner M Zalckvar E. Incredibly close-A newly identified peroxisome-ER contact site in humans. J Cell Biol. (2017) 216:287–9. doi: 10.1083/jcb.201701072 PMC5294797 28108527

[63] Schrader M S Kamoshita M Islinger M. Organelle interplay-peroxisome interactions in health and disease. J Inherit Metab Dis. (2020) 43:71–89. doi: 10.1002/jimd.12083 30864148 PMC7041636

[64] Hua R Cheng D Coyaud E Freeman S Di Pietro E Wang Y. VAPs and ACBD5 tether peroxisomes to the ER for peroxisome maintenance and lipid homeostasis. J Cell Biol. (2017) 216:367–77. doi: 10.1083/jcb.201608128 PMC5294787 28108526

[65] Xiao J Luo J Hu A Xiao T Li M Kong Z. Cholesterol transport through the peroxisome-ER membrane contacts tethered by PI(4,5)P(2) and extended synaptotagmins. Sci China Life Sci. (2019) 62:1117–35. doi: 10.1007/s11427-019-9569-9 31144242

[66] Akbari M Kirkwood T Bohr VA. Mitochondria in the signaling pathways that control longevity and health span. Ageing Res Rev. (2019) 54:100940. doi: 10.1016/j.arr.2019.100940 31415807 PMC7479635

[67] Varabyova A Stojanovski D Chacinska A. Mitochondrial protein homeostasis. IUBMB Life. (2013) 65:191–201. doi: 10.1002/iub.1122 23341326

[68] Han Y Liu D Cheng Y Ji Q Liu M Zhang B. Maintenance of mitochondrial homeostasis for Alzheimer’s disease: Strategies and challenges. Redox Biol. (2023) 63:102734. doi: 10.1016/j.redox.2023.102734 37159984 PMC10189488

[69] Murata D Arai K Iijima M Sesaki H. Mitochondrial division, fusion and degradation. J Biochem. (2020) 167:233–41. doi: 10.1093/jb/mvz106 PMC7048076 31800050

[70] Tilokani L Nagashima S Paupe V Prudent J. Mitochondrial dynamics: overview of molecular mechanisms. Essays Biochem. (2018) 62:341–60. doi: 10.1042/EBC20170104 PMC6056715 30030364

[71] Fung TS Chakrabarti R Higgs HN. The multiple links between actin and mitochondria. Nat Rev Mol Cell Biol. (2023) 24:651–67. doi: 10.1038/s41580-023-00632-9 PMC10528321 37277471

[72] Nagashima S Tabara LC Tilokani L Paupe V Anand H Pogson JH. Golgi-derived PI(4)P-containing vesicles drive late steps of mitochondrial division. Science. (2020) 367:1366–71. doi: 10.1126/science.aax6089 32193326

[73] Wong YC Kim S Peng W Krainc D. Regulation and function of mitochondria-lysosome membrane contact sites in cellular homeostasis. Trends Cell Biol. (2019) 29:500–13. doi: 10.1016/j.tcb.2019.02.004 PMC8475646 30898429

[74] Cheng XT Xie YX Zhou B Huang N Farfel-Becker T Sheng ZH. Revisiting LAMP1 as a marker for degradative autophagy-lysosomal organelles in the nervous system. Autophagy. (2018) 14:1472–4. doi: 10.1080/15548627.2018.1482147 PMC6103665 29940787

[75] Giacomello M Pyakurel A Glytsou C Scorrano L. The cell biology of mitochondrial membrane dynamics. Nat Rev Mol Cell Biol. (2020) 21:204–24. doi: 10.1038/s41580-020-0210-7 32071438

[76] Chan DC. Mitochondrial dynamics and its involvement in disease. Annu Rev Pathol. (2020) 15:235–59. doi: 10.1146/annurev-pathmechdis-012419-032711 31585519

[77] O Oshima Y Verhoeven N Cartier E Karbowski M. The OMM-severed and IMM-ubiquitinated mitochondria are intermediates of mitochondrial proteotoxicity-induced autophagy in PRKN/parkin-deficient cells. Autophagy. (2021) 17:3884–6. doi: 10.1080/15548627.2021.1964887 PMC8632337 34486484

[78] V Valm AM Cohen S Legant WR Melunis J Hershberg U Wait E. Applying systems-level spectral imaging and analysis to reveal the organelle interactome. Nature. (2017) 546:162–7. doi: 10.1038/nature22369 PMC5536967 28538724

[79] Frieden M Arnaudeau S Castelbou C Demaurex N. Subplasmalemmal mitochondria modulate the activity of plasma membrane Ca2+-ATPases. J Biol Chem. (2005) 280:43198–208. doi: 10.1074/jbc.M510279200 16216868

[80] Westermann B. The mitochondria-plasma membrane contact site. Curr Opin Cell Biol. (2015) 35:1–6. doi: 10.1016/j.ceb.2015.03.001 25801776

[81] Szymanski J Janikiewicz J Michalska B Patalas-Krawczyk P Perrone M Ziolkowski W. Interaction of mitochondria with the endoplasmic reticulum and plasma membrane in calcium homeostasis, lipid trafficking and mitochondrial structure. Int J Mol Sci. (2017) 18:1576. doi: 10.3390/ijms18071576 28726733 PMC5536064

[82] Demers-Lamarche J Guillebaud G Tlili M Todkar K Belanger N Grondin M. Loss of mitochondrial function impairs lysosomes. J Biol Chem. (2016) 291:10263–76. doi: 10.1074/jbc.M115.695825 PMC4858975 26987902

[83] Monteleon CL Agnihotri T Dahal A Liu M Rebecca VW Beatty GL. Lysosomes support the degradation, signaling, and mitochondrial metabolism necessary for human epidermal differentiation. J Invest Dermatol. (2018) 138:1945–54. doi: 10.1016/j.jid.2018.02.035 PMC6521870 29526763

[84] Fransen M Lismont C Walton P. The peroxisome-mitochondria connection: how and why? Int J Mol Sci. (2017) 18:1126. doi: 10.3390/ijms18061126 28538669 PMC5485950

[85] White WL. Erratum to: Why I hate the index finger. Handb (N Y). (2011) 6:233. doi: 10.1007/s11552-011-9321-0 PMC3092884 21776199

[86] Rakotonirina-Ricquebourg R Costa V Teixeira V. Hello from the other side: Membrane contact of lipid droplets with other organelles and subsequent functional implications. Prog Lipid Res. (2022) 85:101141. doi: 10.1016/j.plipres.2021.101141 34793861

[87] Cohen S. Lipid droplets as organelles. Int Rev Cell Mol Biol. (2018) 337:83–110. doi: 10.1016/bs.ircmb.2017.12.007 29551163 PMC6241319

[88] Olzmann JA Carvalho P. Dynamics and functions of lipid droplets. Nat Rev Mol Cell Biol. (2019) 20:137–55. doi: 10.1038/s41580-018-0085-z PMC6746329 30523332

[89] Choudhary V Schneiter R. A unique junctional interface at contact sites between the endoplasmic reticulum and lipid droplets. Front Cell Dev Biol. (2021) 9:650186. doi: 10.3389/fcell.2021.650186 33898445 PMC8060488

[90] Combot Y Salo VT Chadeuf G Holtta M Ven K Pulli I. Seipin localizes at endoplasmic-reticulum-mitochondria contact sites to control mitochondrial calcium import and metabolism in adipocytes. Cell Rep. (2022) 38:110213. doi: 10.1016/j.celrep.2021.110213 35021082

[91] Sui X Arlt H Brock KP Lai ZW DiMaio F Marks DS. Cryo-electron microscopy structure of the lipid droplet-formation protein seipin. J Cell Biol. (2018) 217:4080–91. doi: 10.1083/jcb.201809067 PMC6279392 30327422

[92] Salo VT Li S Vihinen H Holtta-Vuori M Szkalisity A Horvath P. Seipin facilitates triglyceride flow to lipid droplet and counteracts droplet ripening via endoplasmic reticulum contact. Dev Cell. (2019) 50:478–93. doi: 10.1016/j.devcel.2019.05.016 31178403

[93] Du X Zhou L Aw YC Mak HY Xu Y Rae J. ORP5 localizes to ER-lipid droplet contacts and regulates the level of PI(4)P on lipid droplets. J Cell Biol. (2020) 219(1):e201905162. doi: 10.1083/jcb.201905162 31653673 PMC7039201

[94] Salo VT Ikonen E. Moving out but keeping in touch: contacts between endoplasmic reticulum and lipid droplets. Curr Opin Cell Biol. (2019) 57:64–70. doi: 10.1016/j.ceb.2018.11.002 30476754

[95] Xu D Li Y Wu L Li Y Zhao D Yu J. Rab18 promotes lipid droplet (LD) growth by tethering the ER to LDs through SNARE and NRZ interactions. J Cell Biol. (2018) 217:975–95. doi: 10.1083/jcb.201704184 PMC5839781 29367353

[96] Xu N Zhang SO Cole RA McKinney SA Guo F Haas JT. The FATP1-DGAT2 complex facilitates lipid droplet expansion at the ER-lipid droplet interface. J Cell Biol. (2012) 198:895–911. doi: 10.1083/jcb.201201139 22927462 PMC3432760

[97] B Benador IY Veliova M Mahdaviani K Petcherski A Wikstrom JD Assali EA. Mitochondria bound to lipid droplets have unique bioenergetics, composition, and dynamics that support lipid droplet expansion. Cell Metab. (2018) 27:869–85. doi: 10.1016/j.cmet.2019.02.011 PMC5969538 29617645

[98] Benador IY Veliova M Mahdaviani K Petcherski A Wikstrom JD Assali EA. Mitochondria bound to lipid droplets have unique bioenergetics, composition, and dynamics that support lipid droplet expansion. Cell Metab. (2018) 27:869–885.e6. doi: 10.1016/j.cmet.2018.03.003 29617645 PMC5969538

[99] Boutant M B Kulkarni SS Joffraud M Ratajczak J Valera-Alberni M Combe R. Mfn2 is critical for brown adipose tissue thermogenic function. EMBO J. (2017) 36:1543–58. doi: 10.15252/embj.201694914 PMC5452040 28348166

[100] Wang J Fang N Xiong J Du Y Cao Y Ji WK. An ESCRT-dependent step in fatty acid transfer from lipid droplets to mitochondria through VPS13D-TSG101 interactions. Nat Commun. (2021) 12:1252. doi: 10.1038/s41467-021-21525-5 33623047 PMC7902631

[101] Titorenko VI Terlecky SR. Peroxisome metabolism and cellular aging. Traffic. (2011) 12:252–9. doi: 10.1111/j.1600-0854.2010.01144.x PMC3077116 21083858

[102] Chang CL Weigel AV Ioannou MS Pasolli HA Xu CS Peale DR. Spastin tethers lipid droplets to peroxisomes and directs fatty acid trafficking through ESCRT-III. J Cell Biol. (2019) 218:2583–99. doi: 10.1083/jcb.201902061 PMC6683741 31227594

[103] Arruda AP Pers BM Parlakgul G Guney E Inouye K Hotamisligil GS. Chronic enrichment of hepatic endoplasmic reticulum-mitochondria contact leads to mitochondrial dysfunction in obesity. Nat Med. (2014) 20:1427–35. doi: 10.1038/nm.3735 PMC4412031 25419710

[104] Feriod CN Oliveira AG Guerra MT Nguyen L Richards KM Jurczak MJ. Hepatic inositol 1,4,5 trisphosphate receptor type 1 mediates fatty liver. Hepatol Commun. (2017) 1:23–35. doi: 10.1002/hep4.1012 28966992 PMC5613674

[105] Wang J He W Tsai PJ Chen PH Ye M Guo J. Mutual interaction between endoplasmic reticulum and mitochondria in nonalcoholic fatty liver disease. Lipids Health Dis. (2020) 19:72. doi: 10.1186/s12944-020-01210-0 32284046 PMC7155254

[106] Madreiter-Sokolowski CT Thomas C Ristow M. Interrelation between ROS and Ca(2+) in aging and age-related diseases. Redox Biol. (2020) 36:101678. doi: 10.1016/j.redox.2020.101678 32810740 PMC7451758

[107] Ziegler DV Vindrieux D Goehrig D Jaber S Collin G Griveau A. Calcium channel ITPR2 and mitochondria-ER contacts promote cellular senescence and aging. Nat Commun. (2021) 12:720. doi: 10.1038/s41467-021-20993-z 33526781 PMC7851384

[108] Beaulant A Dia M Pillot B Chauvin MA Ji-Cao J Durand C. Endoplasmic reticulum-mitochondria miscommunication is an early and causal trigger of hepatic insulin resistance and steatosis. J Hepatol. (2022) 77:710–22. doi: 10.1016/j.jhep.2022.03.017 35358616

[109] Barazzuol L Giamogante F Brini M Cali T. PINK1/parkin mediated mitophagy, ca (2+) signalling, and ER-mitochondria contacts in parkinson’s disease. Int J Mol Sci. (2020) 21:1772. doi: 10.3390/ijms21051772 32150829 PMC7084677

[110] Gelmetti V De Rosa P Torosantucci L Marini ES Romagnoli A Di Rienzo M. PINK1 and BECN1 relocalize at mitochondria-associated membranes during mitophagy and promote ER-mitochondria tethering and autophagosome formation. Autophagy. (2017) 13:654–69. doi: 10.1080/15548627.2016.1277309 PMC5388214 28368777

[111] Zhou P Xie W Meng X Zhai Y Dong X Zhang X. Notoginsenoside R1 ameliorates diabetic retinopathy through PINK1-dependent activation of mitophagy. Cells-Basel. (2019) 8:213. doi: 10.3390/cells8030213 PMC6468581 30832367

[112] Pickles S Vigie P Youle RJ. Mitophagy and quality control mechanisms in mitochondrial maintenance. Curr Biol. (2018) 28:R170–85. doi: 10.1016/j.cub.2018.01.004 PMC7255410 29462587

[113] Liu P Lin H Xu Y Zhou F Wang J Liu J. Frataxin-mediated PINK1-parkin-dependent mitophagy in hepatic steatosis: the protective effects of quercetin. Mol Nutr Food Res. (2018) 62:e1800164. doi: 10.1002/mnfr.201800164 29935106

[114] Li X Shi Z Zhu Y Shen T Wang H Shui G. Cyanidin-3-O-glucoside improves non-alcoholic fatty liver disease by promoting PINK1-mediated mitophagy in mice. Br J Pharmacol. (2020) 177:3591–607. doi: 10.1111/bph.15083 PMC7348088 32343398

[115] Urbina-Varela R Castillo N Videla LA Del CA. Impact of mitophagy and mitochondrial unfolded protein response as new adaptive mechanisms underlying old pathologies: sarcopenia and non-alcoholic fatty liver disease. Int J Mol Sci. (2020) 21(20):7704. doi: 10.3390/ijms21207704 33081022 PMC7589512

[116] Wu S Lu Q Wang Q Ding Y Ma Z Mao X. Binding of FUN14 domain containing 1 with inositol 1,4,5-trisphosphate receptor in mitochondria-associated endoplasmic reticulum membranes maintains mitochondrial dynamics and function in hearts *in vivo* . Circulation. (2017) 136:2248–66. doi: 10.1161/CIRCULATIONAHA.117.030235 PMC5716911 28942427

[117] Wei X Wei X Lu Z Li L Hu Y Sun F. Activation of TRPV1 channel antagonizes diabetic nephropathy through inhibiting endoplasmic reticulum-mitochondria contact in podocytes. Metabolism. (2020) 105:154182. doi: 10.1016/j.metabol.2020.154182 32061660

[118] Wu H Wang Y Li W Chen H Du L Liu D. Deficiency of mitophagy receptor FUNDC1 impairs mitochondrial quality and aggravates dietary-induced obesity and metabolic syndrome. Autophagy. (2019) 15:1882–98. doi: 10.1080/15548627.2019.1596482 PMC6844496 30898010

[119] Gilady SY Bui M Lynes EM Benson MD Watts R Vance JE. Ero1alpha requires oxidizing and normoxic conditions to localize to the mitochondria-associated membrane (MAM). Cell Stress Chaperones. (2010) 15:619–29. doi: 10.1007/s12192-010-0174-1 PMC3006622 20186508

[120] Wright KD Staruschenko A Sorokin A. Role of adaptor protein p66Shc in renal pathologies. Am J Physiol Renal Physiol. (2018) 314:F143–53. doi: 10.1152/ajprenal.00414.2017 PMC5866456 28978535

[121] Chen Z Tian R She Z Cai J Li H. Role of oxidative stress in the pathogenesis of nonalcoholic fatty liver disease. Free Radic Biol Med. (2020) 152:116–41. doi: 10.1016/j.freeradbiomed.2020.02.025 32156524

[122] Song K Zhang Y Ga Q Bai Z Ge RL. High-altitude chronic hypoxia ameliorates obesity-induced non-alcoholic fatty liver disease in mice by regulating mitochondrial and AMPK signaling. Life Sci. (2020) 252:117633. doi: 10.1016/j.lfs.2020.117633 32289432

[123] Campisi J D’Adda DFF. Cellular senescence: when bad things happen to good cells. Nat Rev Mol Cell Biol. (2007) 8:729–40. doi: 10.1038/nrm2233 17667954

[124] Spinelli R S Baboota RK Gogg S Beguinot F Bluher M Nerstedt A. Increased cell senescence in human metabolic disorders. J Clin Invest. (2023) 133:e169922. doi: 10.1172/JCI169922 37317964 PMC10266774

[125] Janikiewicz J Szymanski J Malinska D Patalas-Krawczyk P Michalska B Duszynski J. Mitochondria-associated membranes in aging and senescence: structure, function, and dynamics. Cell Death Dis. (2018) 9:332. doi: 10.1038/s41419-017-0105-5 29491385 PMC5832430

[126] Hetz C Zhang K Kaufman RJ. Mechanisms, regulation and functions of the unfolded protein response. Nat Rev Mol Cell Biol. (2020) 21:421–38. doi: 10.1038/s41580-020-0250-z PMC8867924 32457508

[127] Takeda K Nagashima S Shiiba I Uda A Tokuyama T Ito N. MITOL prevents ER stress-induced apoptosis by IRE1alpha ubiquitylation at ER-mitochondria contact sites. EMBO J. (2019) 38:e100999. doi: 10.15252/embj.2018100999 31368599 PMC6669929

[128] Munoz JP Ivanova S Sanchez-Wandelmer J Martinez-Cristobal P Noguera E Sancho A. Mfn2 modulates the UPR and mitochondrial function via repression of PERK. EMBO J. (2013) 32:2348–61. doi: 10.1038/emboj.2013.168 PMC3770335 23921556

[129] Ngoh GA Papanicolaou KN Walsh K. Loss of mitofusin 2 promotes endoplasmic reticulum stress. J Biol Chem. (2012) 287:20321–32. doi: 10.1074/jbc.M112.359174 PMC3370214 22511781

[130] Han MS Jung DY Morel C Lakhani SA Kim JK Flavell RA. JNK expression by macrophages promotes obesity-induced insulin resistance and inflammation. Science. (2013) 339:218–22. doi: 10.1126/science.1227568 PMC3835653 23223452

[131] Horton JD Goldstein JL Brown MS. SREBPs: activators of the complete program of cholesterol and fatty acid synthesis in the liver. J Clin Invest. (2002) 109:1125–31. doi: 10.1172/JCI15593 PMC150968 11994399

[132] Chen FJ Yin Y Chua BT Li P. CIDE family proteins control lipid homeostasis and the development of metabolic diseases. Traffic. (2020) 21:94–105. doi: 10.1111/tra.12717 31746121

[133] Milic S Lulic D Stimac D. Non-alcoholic fatty liver disease and obesity: biochemical, metabolic and clinical presentations. World J Gastroenterol. (2014) 20:9330–7. doi: 10.3748/wjg.v20.i28.9330 PMC4110564 25071327

[134] Suzuki M. Regulation of lipid metabolism via a connection between the endoplasmic reticulum and lipid droplets. Anat Sci Int. (2017) 92:50–4. doi: 10.1007/s12565-016-0378-2 27822589

[135] Krahmer N Najafi B Schueder F Quagliarini F Steger M Seitz S. Organellar proteomics and phospho-proteomics reveal subcellular reorganization in diet-induced hepatic steatosis. Dev Cell. (2018) 47:205–21. doi: 10.1016/j.devcel.2018.09.017 30352176

[136] Keenan SN Meex RC Lo J Ryan A Nie S Montgomery MK. Perilipin 5 deletion in hepatocytes remodels lipid metabolism and causes hepatic insulin resistance in mice. Diabetes. (2019) 68:543–55. doi: 10.2337/db18-0670 30617219

[137] Li H Song Y Zhang LJ Gu Y Li FF Pan SY. LSDP5 enhances triglyceride storage in hepatocytes by influencing lipolysis and fatty acid beta-oxidation of lipid droplets. Plos One (2012) 7(6):e36712. doi: 10.1371/journal.pone.0036712 22675471 PMC3365886

[138] Tan Y Jin Y Wang Q Huang J Wu X Ren Z. Perilipin 5 protects against cellular oxidative stress by enhancing mitochondrial function in hepG2 cells. Cells-Basel. (2019) 8:1241. doi: 10.3390/cells8101241 PMC6830103 31614673

[139] Wang C Zhao Y Gao X Li L Yuan Y Liu F. Perilipin 5 improves hepatic lipotoxicity by inhibiting lipolysis. Hepatology. (2015) 61:870–82. doi: 10.1002/hep.27409 25179419

[140] Varghese M Kimler VA Ghazi FR Rathore GK Perkins GA Ellisman MH. Adipocyte lipolysis affects Perilipin 5 and cristae organization at the cardiac lipid droplet-mitochondrial interface. Sci Rep. (2019) 9:4734. doi: 10.1038/s41598-019-41329-4 30894648 PMC6426865

[141] Schuldiner M S Bohnert M. A different kind of love - lipid droplet contact sites. Biochim Biophys Acta Mol Cell Biol Lipids. (2017) 1862:1188–96. doi: 10.1016/j.bbalip.2017.06.005 28627434

[142] Filali-Mouncef Y Hunter C Roccio F Zagkou S Dupont N Primard C. The menage a trois of autophagy, lipid droplets and liver disease. Autophagy. (2022) 18:50–72. doi: 10.1080/15548627.2021.1895658 33794741 PMC8865253

[143] Mao H Chen W Chen L Li L. Potential role of mitochondria-associated endoplasmic reticulum membrane proteins in diseases. Biochem Pharmacol. (2022) 199:115011. doi: 10.1016/j.bcp.2022.115011 35314166

[144] Rieusset J. Endoplasmic reticulum-mitochondria calcium signaling in hepatic metabolic diseases. Biochim Biophys Acta Mol Cell Res. (2017) 1864:865–76. doi: 10.1016/j.bbamcr.2017.01.001 28064001

[145] Jin C Kumar P Gracia-Sancho J Dufour JF. Calcium transfer between endoplasmic reticulum and mitochondria in liver diseases. FEBS Lett. (2021) 595:1411–21. doi: 10.1002/1873-3468.14078 33752262

[146] Zhang J Li Y Jiang S Yu H An W. Enhanced endoplasmic reticulum SERCA activity by overexpression of hepatic stimulator substance gene prevents hepatic cells from ER stress-induced apoptosis. Am J Physiol Cell Physiol. (2014) 306:C279–90. doi: 10.1152/ajpcell.00117.2013 24284796

[147] Lai S Li Y Kuang Y Cui H Yang Y Sun W. PKCdelta silencing alleviates saturated fatty acid induced ER stress by enhancing SERCA activity. Biosci Rep. (2017) 37:BSR20170869. doi: 10.1042/BSR20170869 29046367 PMC5700272

[148] Shen ZQ Chen YF Chen JR Jou YS Wu PC Kao CH. CISD2 haploinsufficiency disrupts calcium homeostasis, causes nonalcoholic fatty liver disease, and promotes hepatocellular carcinoma. Cell Rep. (2017) 21:2198–211. doi: 10.1016/j.celrep.2017.10.099 29166610

[149] Coen K Flannagan RS Baron S Carraro-Lacroix LR Wang D Vermeire W. Lysosomal calcium homeostasis defects, not proton pump defects, cause endo-lysosomal dysfunction in PSEN-deficient cells. J Cell Biol. (2012) 198:23–35. doi: 10.1083/jcb.201201076 22753898 PMC3392942

[150] Fernandez-Mosquera L Yambire KF Couto R Pereyra L Pabis K Ponsford AH. Mitochondrial respiratory chain deficiency inhibits lysosomal hydrolysis. Autophagy. (2019) 15:1572–91. doi: 10.1080/15548627.2019.1586256 PMC6693470 30917721

[151] Cavinato M Madreiter-Sokolowski CT Buttner S Schosserer M Zwerschke W Wedel S. Targeting cellular senescence based on interorganelle communication, multilevel proteostasis, and metabolic control. FEBS J. (2021) 288:3834–54. doi: 10.1111/febs.15631 PMC7611050 33200494

[152] Atakpa P Thillaiappan NB Mataragka S Prole DL Taylor CW. IP(3) receptors preferentially associate with ER-lysosome contact sites and selectively deliver ca(2+) to lysosomes. Cell Rep. (2018) 25:3180–93. doi: 10.1016/j.celrep.2018.11.064 PMC6302550 30540949

[153] Ghislat G Knecht E. Ca(2)(+)-sensor proteins in the autophagic and endocytic traffic. Curr Protein Pept Sci. (2013) 14:97–110. doi: 10.2174/13892037112139990033 23305313 PMC3664516

[154] Madureira M Connor-Robson N Wade-Martins R. LRRK2: autophagy and lysosomal activity. Front Neurosci. (2020) 14:498. doi: 10.3389/fnins.2020.00498 32523507 PMC7262160

[155] Fujii S Hara H Araya J Takasaka N Kojima J Ito S. Insufficient autophagy promotes bronchial epithelial cell senescence in chronic obstructive pulmonary disease. Oncoimmunology. (2012) 1:630–41. doi: 10.4161/onci.20297 PMC3429567 22934255

[156] Korolchuk VI Miwa S Carroll B von Zglinicki T. Mitochondria in cell senescence: is mitophagy the weakest link? Ebiomedicine. (2017) 21:7–13. doi: 10.1016/j.ebiom.2017.03.020 28330601 PMC5514379

[157] Zhang X Cheng X Yu L Yang J Calvo R Patnaik S. MCOLN1 is a ROS sensor in lysosomes that regulates autophagy. Nat Commun. (2016) 7:12109. doi: 10.1038/ncomms12109 27357649 PMC4931332

[158] Di Paola S Scotto-Rosato A Medina DL. TRPML1: the ca((2+))retaker of the lysosome. Cell Calcium. (2018) 69:112–21. doi: 10.1016/j.ceca.2017.06.006 28689729

[159] Santoni G Amantini C Nabissi M Maggi F Arcella A Marinelli O. Knock-down of mucolipin 1 channel promotes tumor progression and invasion in human glioblastoma cell lines. Front Oncol. (2021) 11:578928. doi: 10.3389/fonc.2021.578928 33954107 PMC8092188

[160] Longo M Meroni M Paolini E Macchi C Dongiovanni P. Mitochondrial dynamics and nonalcoholic fatty liver disease (NAFLD): new perspectives for a fairy-tale ending? Metabolism. (2021) 117:154708. doi: 10.1016/j.metabol.2021.154708 33444607

[161] Begriche K Massart J Robin MA Bonnet F Fromenty B. Mitochondrial adaptations and dysfunctions in nonalcoholic fatty liver disease. Hepatology. (2013) 58:1497–507. doi: 10.1002/hep.26226 23299992

[162] Pernas L Scorrano L. Mito-morphosis: mitochondrial fusion, fission, and cristae remodeling as key mediators of cellular function. Annu Rev Physiol. (2016) 78:505–31. doi: 10.1146/annurev-physiol-021115-105011 26667075

[163] Afonso MB Islam T Magusto J Amorim R Lenoir V Simoes RF. RIPK3 dampens mitochondrial bioenergetics and lipid droplet dynamics in metabolic liver disease. Hepatology. (2023) 77:1319–34. doi: 10.1002/hep.32756 PMC10026966 36029129

[164] Gancheva S Kahl S Pesta D Mastrototaro L Dewidar B Strassburger K. Impaired hepatic mitochondrial capacity in nonalcoholic steatohepatitis associated with type 2 diabetes. Diabetes Care. (2022) 45:928–37. doi: 10.2337/dc21-1758 35113139

[165] Shin S Kim J Lee JY Kim J Oh CM. Mitochondrial quality control: its role in metabolic dysfunction-associated steatotic liver disease (MASLD). J Obes Metab Syndr. (2023) 32:289–302. doi: 10.7570/jomes23054 38049180 PMC10786205

[166] Mishra P Chan DC. Metabolic regulation of mitochondrial dynamics. J Cell Biol. (2016) 212:379–87. doi: 10.1083/jcb.201511036 PMC4754720 26858267

[167] Sookoian S Flichman D Scian R Rohr C Dopazo H Gianotti TF. Mitochondrial genome architecture in non-alcoholic fatty liver disease. J Pathol. (2016) 240:437–49. doi: 10.1002/path.4803 27577682

[168] Akie TE Liu L Nam M Lei S Cooper MP. OXPHOS-mediated induction of NAD+ Promotes complete oxidation of fatty acids and interdicts non-alcoholic fatty liver disease. PloS One. (2015) 10:e125617. doi: 10.1371/journal.pone.0125617 PMC4416931 25933096

[169] Childs BG Durik M Baker DJ van Deursen JM. Cellular senescence in aging and age-related disease: from mechanisms to therapy. Nat Med. (2015) 21:1424–35. doi: 10.1038/nm.4000 PMC4748967 26646499

